# Evidence for synovial fibroblasts spreading rheumatoid arthritis

**DOI:** 10.1186/ar3570

**Published:** 2012-02-09

**Authors:** Ulf Müller-Ladner, Stefanie Lefèvre, Birgit Zimmermann, Ingo H Tarner, Robert Dinser, Thomas Pap, Steffen Gay, Elena Neumann

**Affiliations:** 1 Dept Internal Medicine and Rheumatology, JLU Giessen, Kerckhoff-Clinic, Bad Nauheim, 61231 Germany; 2 Div Mol Med of Musculoskeletal Tissue, University Hospital Münster, Germany; 3 Ctr Exp Rheumatology, Zürich Center for Integrative Human Physiology, USZ, Zürich, 8006 Switzerland

## Background

Active rheumatoid arthritis (RA) is characterized by continuous progression of the inflammatory process, eventually affecting the majority of joints. Thus far, molecular and cellular pathways of disease progression are largely unknown. One of the key players in this destructive scenario are synovial fibroblasts (SF) which actively attach to, invade into and degrade articular cartilage. As RASF are able to migrate *in vitro*, the current series of experiments were designed to evaluate the potential of RASF to spread the disease *in vivo *in the SCID mouse model of RA.

## Methods

Healthy human cartilage was co-implanted subcutaneously into SCID mice together with RASF. At the contralateral flank, simulating an unaffected joint, cartilage was implanted without cells. To analyze the route of migration of RASF, the cells were injected subcutaneously, intraperitoneally or intravenously before or after implantation of cartilage. In addition, whole RA synovium and normal human cartilage were implanted separately in order to analyze the effects of matrix and other cells on the migratory behavior of RASF. To evaluate potential influences of wound healing, either the primary RASF-containing implant or the contralateral implant without RASF, respectively, was inserted first, followed by implantation of the corresponding other implant after 14 days. After 60 days, implants, organs and blood were removed and analyzed. For the detection of human cells, immunohisto- and -cytochemistry were performed with species-specific antibodies.

## Results

RASF not only invaded and degraded the co-implanted cartilage, they also migrated to and invaded into the contralateral cell free implanted cartilage. Injection of RASF led to a strong destruction of the implanted cartilage, particularly after subcutaneous and intravenous application. Interestingly, implantation of whole synovial tissue also resulted in migration of RASF to the contralateral cartilage in one third of the animals. With regard to the route of migration, few RASF could be detected in spleen, heart and lung, mainly located in vessels, most likely resulting from an active movement to the target cartilage *via *the vasculature. With respect to functional aspects, growth factors and adhesion molecules appear to influence significantly the migratory behavior of the synovial fibroblasts.

## Conclusions

The results support the hypothesis that the clinically characteristic phenomenon of inflammatory spreading from joint to joint is mediated, at least in part, by a transmigration of activated RASF, regulated by growth factors and adhesion molecules.

